# Bone Regeneration by Novel Bioactive Glasses Containing Strontium and/or Magnesium: A Preliminary In-Vivo Study

**DOI:** 10.3390/ma11112223

**Published:** 2018-11-08

**Authors:** Devis Bellucci, Valeria Cannillo, Alexandre Anesi, Roberta Salvatori, Luigi Chiarini, Tiziano Manfredini, Davide Zaffe

**Affiliations:** 1 Department of Engineering “E. Ferrari”, University of Modena and Reggio Emilia, Via P. Vivarelli 10, 41125 Modena, Italy; valeria.cannillo@unimore.it (V.C.); tiziano.manfredini@unimore.it (T.M.); 2 Laboratory of Biomaterials, Department of Medical and Surgical Sciences of Children & Adults, University of Modena and Reggio Emilia, Via Campi 213/A, 41125 Modena, Italy; alexandre.anesi@unimore.it (A.A.); roberta.salvatori@unimore.it (R.S.); luigi.chiarini@unimore.it (L.C.); 3 Department of Biomedical, Metabolic and Neural Sciences, University of Modena and Reggio Emilia, 41125 Modena, Italy; davide.zaffe@unimore.it

**Keywords:** bioactive glasses, in vivo study, regenerative medicine, bone regeneration, strontium, magnesium

## Abstract

In this work, a set of novel bioactive glasses have been tested in vivo in an animal model. The new compositions, characterized by an exceptional thermal stability and high in vitro bioactivity, contain strontium and/or magnesium, whose biological benefits are well documented in the literature. To simulate a long-term implant and to study the effect of the complete dissolution of glasses, samples were implanted in the mid-shaft of rabbits’ femur and analyzed 60 days after the surgery; such samples were in undersized powder form. The statistical significance with respect to the type of bioactive glass was analyzed by Kruskal–Wallis test. The results show high levels of bone remodeling, several new bone formations containing granules of calcium phosphate (sometimes with amounts of strontium and/or magnesium), and the absence of adverse effects on bone processes due to the almost complete glass dissolution. In vivo results confirming the cell culture outcomes of a previous study highlighted that these novel bioglasses had osteostimulative effect without adverse skeletal reaction, thus indicating possible beneficial effects on bone formation processes. The presence of strontium in the glasses seems to be particularly interesting.

## 1. Introduction

The demand for new materials suitable for healing of tissues and bone degeneration pathologies is dramatically increasing because of world population ageing. Pathologies associated with the musculoskeletal system affect hundreds of millions of people all over the world [1,2,3,4]. In the last decades, several studies attempted to produce high performance materials with enhanced biological response. In this context, the interest of materials’ scientists has focused on calcium phosphate ceramics (CPs), which currently play a primary role in orthopedics, hand surgery, and maxillofacial and oral surgery [2]. Thanks to their high biocompatibility, CPs are perfect candidates to manufacture prostheses, orthopedic devices and synthetic bone grafts. Among CPs, hydroxyapatite (HA) is probably the most widely used material, by virtue of its close crystal and chemical similarity to the inorganic component of biological hard tissues (bone and teeth). In fact, HA is highly osteoconductive and is able to form a strong bond with the surrounding living bone [1].

Bioactive glasses could be an attractive alternative to HA, as they are typically able to bond to bone more rapidly than other bioceramics. Since its discovery, 45S5 Bioglass^®^ (45S5) opened unimaginable scenarios in the field of tissue regeneration, mainly thanks to its osteoinductive and osteoconductive ability [3,4]. Bioactive glasses have shown to have unique properties compared to CPs and HA. In vitro studies reported that, during the dissolution of 45S5, the ion release seems to induce angiogenesis and stimulate osteoblasts proliferation and new bone growth [5]. Moreover, some bioactive glasses are able to bond to both bone and soft connective tissues [6]. However, the 45S5 and in general bioactive glasses show some drawbacks, in particular, their tendency to crystallize during the thermal treatments which are necessary to produce sintered materials, such as scaffolds, coatings, and composites with HA or CPs as a second phase. In fact, crystallization is likely to reduce the bioactivity of the final system and possibly lead to implant instability, as the residual glassy phase is degraded preferentially [4]. Thus, the investigation of new compositions of bioactive glass with low crystallization tendency, to be used whenever a heat treatment is needed, is crucial.

Previous research demonstrated that the so called BG_Ca-Mix (composition, in mol%: 2.3 Na_2_O; 2.3 K_2_O; 45.6 CaO; 2.6 P_2_O_5_; 47.2 SiO_2_), a bioglass developed in recent years, is particularly promising by virtue of both its lower tendency to crystallize with respect to 45S5 and its bioactivity [7,8,9]: in fact, BG_Ca-Mix starts to crystallize at temperatures as high as 880 °C, whilst a crystallization temperature of about 650 °C is reported for 45S5 [10]. In addition, BG_Ca-Mix is characterized by a slower ion leaching in simulated body fluid (SBF) with respect to 45S5, resulting in pH values which are optimal for cell adhesion, proliferation, and differentiation [7].

The feasibility of modified BG_Ca-Mix compositions, containing MgO and/or SrO, for the purpose of combining the favorable thermal stability of the parent BG_Ca-Mix glass with the documented biological benefits of magnesium and strontium, has been recently demonstrated for the first time [11]. In fact, it is well known that magnesium is essential to bone metabolism, stimulates new bone formation, and increases bone cell adhesion [12], while strontium can stimulate osteoblasts and inhibit osteoclasts in vitro, thus it can be exploited to reduce bone resorption and to stimulate osteogenesis in vivo [13]. Moreover, such bioactive glasses with low crystallization temperatures have been successfully employed to realize composites in conjunction with HA, containing various volume fractions of bioactive glass [9,14]. It is worth noting that the production of bioactive glass/HA composites is very interesting, since it is possible to overcome the intrinsic limits of the glassy and the ceramic phases individually. In this way, the material can be designed for a given application, i.e., it is possible to tailor the biodegradation rate and the bioactivity of the resulting system by varying the volume fractions of the two constituents. Thanks to the peculiarities of the novel bioactive glasses, it was possible to sinter the HA-based composites at lower temperatures with respect to samples with the same HA/45S5 ratio, thus reducing the crystallization of the glassy phase and avoiding the decomposition of HA and/or reactions between HA and the glass [8,9].

All the compositions, i.e., BG_Ca-Mix, modified BG_Ca-Mix compositions with MgO and/or SrO and HA/BG_Ca-Mix composites have been successfully tested in vitro in terms of biocompatibility [9,15]. In another very recent paper [16], sintered samples of the BG_Ca-Mix family and specific HA/BG_Ca-Mix composites have been tested in vivo in an animal model, showing promising results.

In this preliminary work, for the first time, three modified BG_Ca-Mix compositions in powder form, each containing MgO and/or SrO, have been tested in vivo in an animal model and compared. The samples were implanted in the mid-shaft of rabbits’ femurs. The implant would simulate a long-term implant, with complete dissolution of the glass, in cortical bone to analyze either glass behavior and bone regeneration processes.

## 2. Materials and Methods

### 2.1. Bioactive Glasses Preparation

The BGMg, BGSr, and BGMgSr bioactive glasses [11], whose final compositions (in mol% oxides) are reported in Table 1, were produced by melting the commercial raw powders (SiO_2_, Ca_3_(PO_4_)_2_, CaCO_3_, Na_2_CO_3_, K_2_CO_3_, SrCO_3_, Mg(OH)_2_·5H_2_O all reagent grade—Carlo Erba Reagenti, Italy) in Pt crucibles at 1450 °C, as previously reported [7,16,17]. The following thermal cycle was employed: from room temperature to 1100 °C at 10 °C/min; an isothermal step at 1100 °C for 1 h to allow decarbonation; from 1100 °C to 1450 °C at 10 °C/min. The molten glasses were quenched into water to obtain the frits, which have been left to dry in an oven at 110 °C for 24 h and subsequently crushed in dry conditions in a porcelain jar. The frits were sieved to obtain glass powders with grain size <63 μm. Finally, an autoclave sterilization of the glass powders was carried out at 121 °C for 20 min.

### 2.2. Animals and Surgery

Experiments were performed according to the Bioethical Committee of the Italian National Institute of Health and authorized with Decrees of the Italian Ministry of Health (Protocol Number: 210/2013-B). Animal care, maintenance, and surgery were conducted in accordance with Italian law (D.L. No. 26/2014) and European legislation (EEC No. 63/2010).

Six healthy six-months-old white New Zealand rabbits (Harlan Laboratories S.r.l., Correzzana MB, Italy) with an average body weight of 5 kg were used. General anesthesia was induced by a mixture of xylazine (4 mg/kg body weight—Sedaxylan, Dechra Veterinary Products S.r.l., Torino, Italy) and ketamine (30 mg/kg body weight—Imalgene 1000, Merial Italia S.p.A., Milano, Italy). If necessary, further sedation was obtained by means of propofol (7 mg/kg—Propovet, Ecuphar S.r.l., Piacenza, Italy) administered in the marginal ear vein.

After induction of anesthesia, shaving and antisepsis were carried out on the legs to be operated. A 3 cm long skin incision was made on the antero-lateral surface of the tight; after blunt dissection of muscles, an incision was made on the periosteum by a scalpel to expose the femur cortex. Bilateral 2.5-mm-diameter bone defects were surgically created at the upper third of the rabbit femur under continuous saline irrigation, using a bone trepan bur under irrigation with saline solution. Holes were compressed with gauze for 5 min, then randomly filled with the appropriate bioactive glass (3 femurs per glass type) or left empty (control). Blood coming from the medullary cavity was mixed to the grafted material (Table 2). Flaps were sutured and rabbits underwent antibiotic and analgesic treatments.

### 2.3. Histology, SEM, and X-ray Microanalysis, Histomorphometric Analysis

Rabbits were euthanized 60 days after surgery by intravenous injection of embutramide/mebezonium iodide (0.3 mL/kg body weight—Tanax 50 mg, MSD Animal Health S.r.l. Italia, Segrate MI, Italy) under general anesthesia with a mixture of xylazine (4 mg/kg body weight) (Sedaxylan^®^, Dechra Veterinary Products Srl, Turin, Italy) and ketamine (30 mg/kg body weight) (Imalgene 1000^®^, Merial Italia SpA, Milan, Italy). The disarticulated femurs were fixed in 4% formaldehyde solution. Femurs were dehydrated in ethanol, radiographed, then methacrylate (PMMA) embedded using a water bath at 4 °C [18].

PMMA femurs were serially sectioned to obtain 300-micron-thick transverse sections of the shaft. After emery paper polishing of surfaces, sections were microradiographed (3K5, Italstructures) at 12 kV and 2 mA on low-resolution film (EM film, Ilford Ltd., Mobberley, Cheshire, UK).

After surface polishing with emery paper and alumina, sections were fastened to aluminium stubs and then examined by scanning electron microscope (SEM Quanta 200, FEI Company, Heindhoven, The Netherlands), equipped with X-ray energy dispersive microscopy chemical microanalysis (EDS INCA-350, Oxford instruments, Abingdon, UK). This microscope allows the analysis of non-conductive surfaces using a low vacuum water saturated conductive atmosphere (low-vacuum scanning electron microscopy—LV-SEM). The analyses were performed at 20 KV, in low vacuum conditions (0.53 Torr), using a solid-state backscattered electron detector. The chemical composition of analyzed surfaces was checked using EDS microanalysis. Semiquantitative analysis was subsequently run after appropriate ZAF (Z, atomic number; A, absorption; F, secondary fluorescence) correction, using proprietary software (INCA Suite version 4.07, Oxford Instruments).

Backscattered SEM images were examined and scored for bone healing process (Table 3). Foreign tissue reaction, osteogenesis, fibrotic tissue growth, physical attachment, biocompatibility, and resorption in the graft criteria were reviewed at histological examination, using modified Emery’s histopathological criteria [19]. Data were analyzed using the Kruskal–Wallis test in order to determine in which group there was a statistical difference in terms of bone healing scores. Since variables were discontinuous, the Kruskal–Wallis test was selected for statistical analysis, with three-way variance and a *p* < 0.05 level of significance. The IBM^®^ SPSS Statistics^®^ package program was used for statistical analyses. Bone regeneration was evaluated by measuring, on digitalized pictures, the amount of bone present inside the gap, and expressed as BV/TV%. The area of bone present inside the osteotomy (BV) and the total area of the osteotomy (TV) were measured using the Image-J ‘polygon tool’, in order to calculate the BV/TV% value [20].

## 3. Results

**Figure 1:**
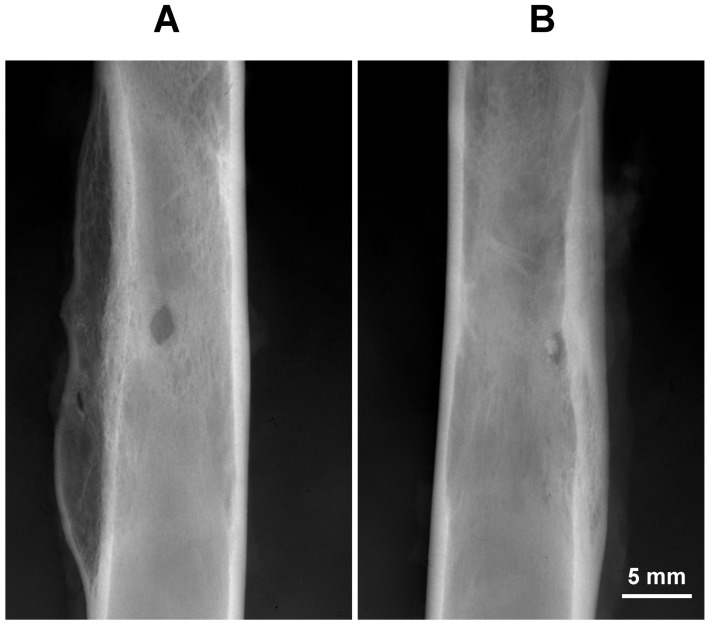
Representative mid-shaft radiographs of two femurs 60 days after the surgery. Note in both radiographs how the shape of hole changes from round (original) to ellipsoidal. Note also how the control hole (**A**) has a greater size than that of a BGSr filled hole (**B**).

The healing of all implanted rabbits was uneventful. Reparative processes changed the regular shape of the holes (Figure 1). Sixty days after surgery, the holes become ellipsoidal with a major longitudinal diameter, sometimes greater than the initial in control holes, and a minor horizontal diameter (Figure 1A). Holes filled with glasses showed reduced size when compared to control holes 60 days after surgery.

**Figure 2:**
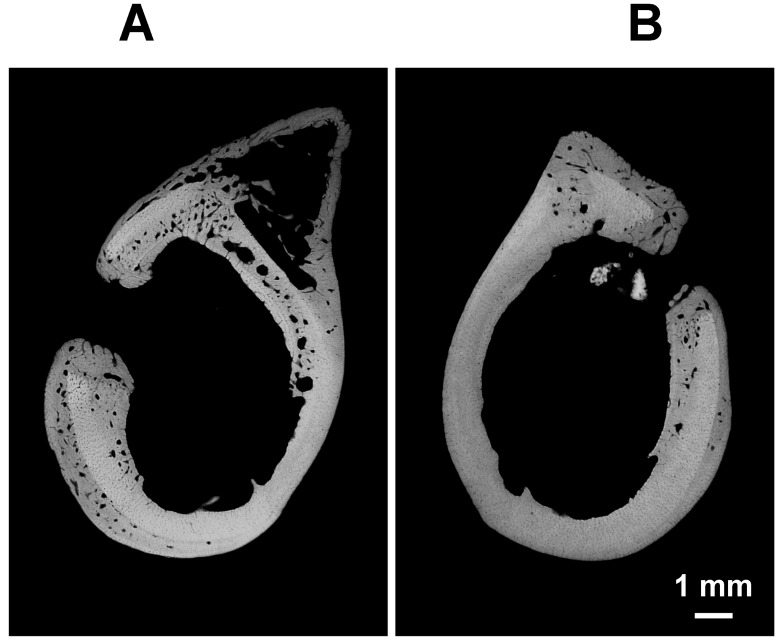
Representative microradiographs of two transverse sections taken at the central part of control hole (**A**) and BGSr filled hole (**B**), 60 days after the surgery. Note in both microradiographs how the hole wall has been greatly remodeled and that new bone (dark gray) now replaces the resorbed pre-existing bone. Note also how the transverse diameter of the control hole (**A**) is almost twice the corresponding diameter of the BgSr filled hole (**B**).

Microradiographs of transverse sections highlighted the presence of a newly formed posterior crest as a result of bone reactivity to the surgical injury (Figure 2). Moreover, the bone of the hole wall was resorbed increasing the transverse diameter up to 3.5 mm (Figure 2). New bone, starting from the hole wall, spared from the osteoclast erosion and formed during the post-surgery time, reduced the final size of the transverse hole diameter to 1–1.5 mm, with lesser size in the glass filled holes (Figure 2). Additional calcified formations were often found in the medullar cavity in proximity of the endosteal boundary of the hole (Figure 2).

**Figure 3:**
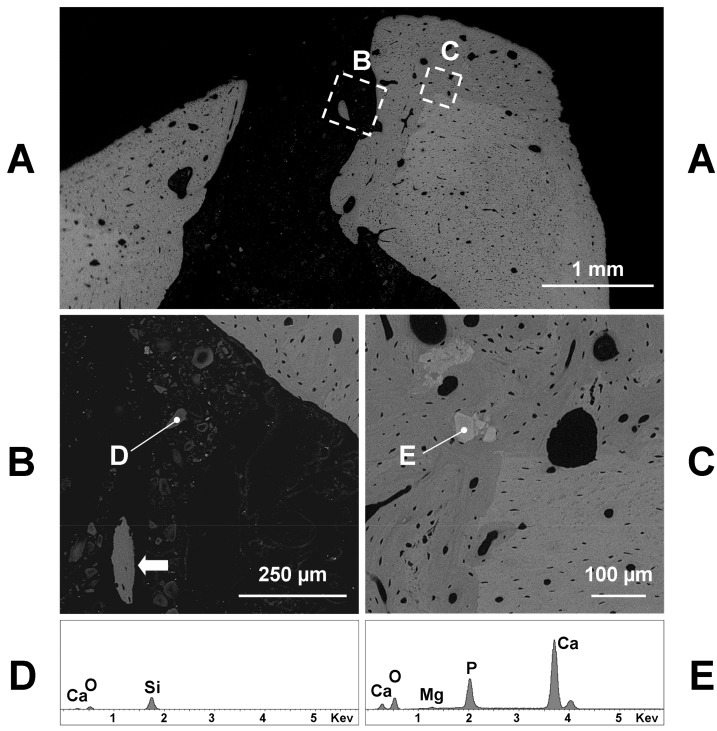
Representative backscattered SEM images of a transverse section taken at the central part of a BGMg filled hole, 60 days after the surgery. The **B** and **C** boxed areas of **A** correspond to the hole and wall image (**B**) and to the remodeled bone image (**C**), at a greater magnification. Note in **B** a small piece of newly formed bone (arrow) inside the hole. Note also in **B** the remnants of the implanted glass, whose X-ray microanalysis (**D**) points to a small fragments of silica gel containing small amounts of calcium. Near the junction with the pre-existing bone (lighter in **C**) some granules (**E**) and several irregularly shaped osteocytes are included in newly formed bone (darker in **C**). The X-ray microanalysis (**E**) highlights granules consist of calcium phosphate with a small amount of magnesium.

**Figure 4:**
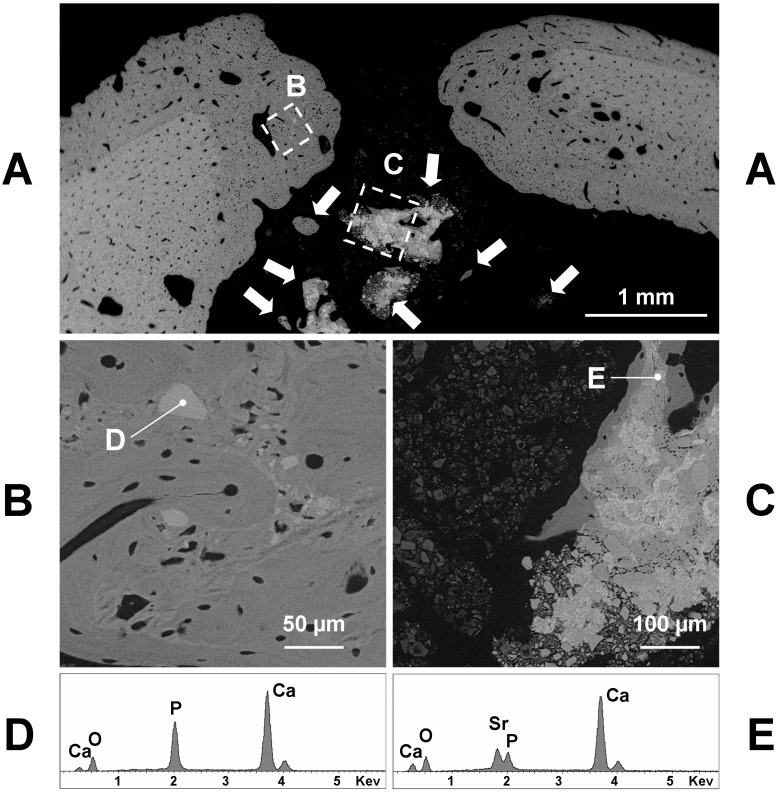
Representative backscattered SEM images of a transverse section taken at the central part of a BGSr filled hole, 60 days after the surgery, showing several isolated bone formations (arrows) inside the medullar cavity. The **B** and **C** boxed areas of **A** correspond to the remodeled bone (**B**) and to an isolated bone formation (**C**), at a greater magnification. The newly formed bone (darker in **A**) contains several irregularly shaped osteocytes (**B**) and includes some granules (**D**) consisting of calcium phosphate, as shown by the X-ray microanalysis (**D**). The isolated bone formations, surrounded by the remnants of the implanted glass (**C**, on the left), often contain mineral granules (lighter in **C**) consisting of calcium phosphate with amounts of strontium, as shown by the X-ray microanalysis (**E**).

**Figure 5:**
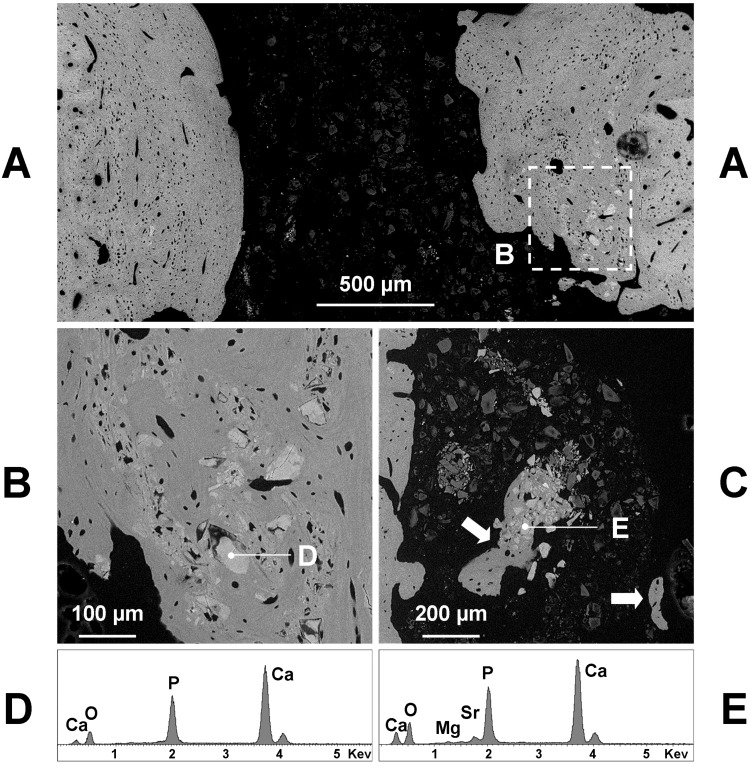
Representative backscattered SEM images of a transverse section taken at the central part of a BGMgSr filled hole, 60 days after the surgery. The **B** boxed area of **A** correspond to the remodeled bone (**B**) at a greater magnification. The newly formed bone (darker in **A**) contains several irregularly shaped osteocytes (**B**) and includes some granules (**D**) consisting of calcium phosphate, as shown by the X-ray microanalysis (**D**). Few isolated bone formations (arrows in **C**) of the medullar cavity are surrounded by remnants of the implanted glass (**C**), sometimes contain mineral granules (**E**, lighter in **C**) consisting of calcium phosphate with amounts of strontium and magnesium, as shown by the X-ray microanalysis (**E**).

The SEM analysis confirmed the microradiographic data, since the hole diameter of glass grafted femurs ranged from 1 to 1.5 mm (Figure 3A, Figure 4A and Figure 5A). If we exclude the initial post-surgical resorption, no sign of cortical bone resorption was observed in all samples containing the implanted glasses 60 days after the surgery. Few remnants of glasses were found inside the hole or the medullar cavity (Figure 3B). They appeared very dark (electron-transparent) at the backscattered detector of SEM and show a size of 20–30 µm or lesser (Figure 3B). The X-ray microanalysis showed that the remnants of BGMg, BGSr, and BGMgSr glasses were similar and almost constituted by silica gel, sometimes containing small amounts of calcium (Figure 3D—98.1% SiO_2_, 1.9% CaO).

The grafting of BGMg produced a good amount of newly formed bone in apposition to the surgically treated pre-existing bone (Figure 3A,C). Sometimes small pieces of newly formed bone were found inside the soft tissue of the hole or in the medullar cavity containing remnants of glasses (Figure 3B). The new bone was almost woven structured with irregularly shaped osteocytes and often it did include some granules near the junction with the pre-existing bone (Figure 3C). These granules had a size of 50–100 µm and appeared lighter (electron-opaque) than the old bone at the backscattered detector of SEM. The X-ray microanalysis showed that calcium phosphate with small amounts of magnesium was the composition of such granules (Figure 3E—62.2% CaO 36.0% P_2_O_5_, 1.8% MgO; Ca/P = 2.19).

Also, the BGSr grafting produced a good amount of newly formed bone in apposition to the pre-existing bone (Figure 4A). The new bone had a woven structure and midmost included 30–50-micron granules (Figure 4B). Such granules appeared lighter (electron-opaque) than the bone at the backscattered detector of SEM and the X-ray microanalysis showed that they were exclusively formed by calcium phosphate (Figure 4D, 55.3% CaO 44.7% P_2_O_5_; Ca/P = 1.57). Some great isolated bone formations, containing or not mineral granules (Figure 4A) but always surrounded by remnants of BGSr glass (Figure 4B), were found inside the soft tissue of the medullar cavity (Figure 4A). The mineral compounds, lighter (electron-opaque) than the bone at the backscattered detector of SEM—on which new bone was formed by apposition to (Figure 4C)—at the X-ray microanalysis resulted constituted by calcium phosphate with amounts of strontium (Figure 4E—58.9% CaO 21.3% P_2_O_5_, 19.8% SrO; Ca/P = 3.5).

The BGMgSr grafting showed outcomes similar to those of either BGMg and BGSr grafting. BGMgSr produced a good amount of newly formed bone in apposition to the pre-existing bone (Figure 5A). The new bone had a woven structure and included 30–80-micron granules near the junction with the pre-existing bone (Figure 5A). These granules appeared lighter (electron-opaque) than the bone at the backscattered detector of SEM and the X-ray microanalysis showed that they were exclusively formed by calcium phosphate (Figure 5D—56.2% CaO 43.8% P_2_O_5_; Ca/P = 2.10). Few isolated bone formations, containing or not mineral granules (Figure 4A) but always surrounded by remnants of BGSr glass (Figure 4B), were found inside the soft tissue of the medullar cavity (Figure 4A). The mineral compounds, lighter (electron-opaque) than the bone at the backscattered detector of SEM, on which new bone was formed by apposition to (Figure 5C), at the X-ray microanalysis resulted constituted by calcium phosphate with small amounts of strontium and magnesium (Figure 5E—51.0% CaO 45.7% P_2_O_5_, 1.9% SrO, 1.4% MgO; Ca/P = 1.93).

**Figure 6:**
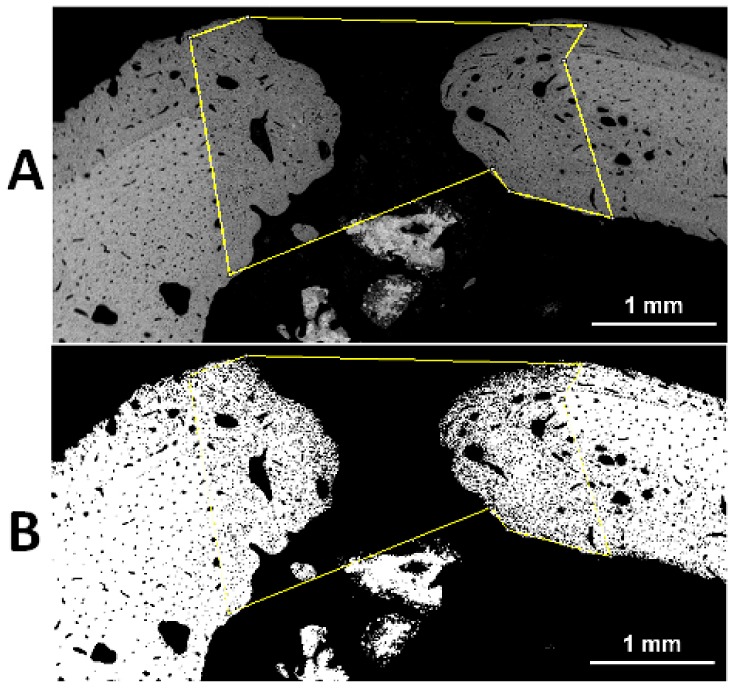
Representative backscattered SEM images of a osteotomy gap of a BGSr sample (see Figure 4A). Bone regeneration was evaluated by measuring, on digitalized pictures, the amount of newly formed bone present inside the gap expressed as BV/TV%. The area of bone present inside the osteotomy (BV) and the total area of the osteotomy (TV) were measured using the Image-J ‘polygon tool’, in order to calculate the BV/TV% value. The yellow polygon outlined the newly formed bone in the osteotomic gap (darker in **A**). The adjusted black & white threshold (**B**) of the Image-J software allows digital measurement of BV/TV% value.

Concerning BV/TV, differences were found between BGMg, BGSr, and BGMgSr samples: the values are higher in BGSr with respect to BGMg and BGMgSr group, although this was not statistically significant because of small sample size (see Table 4, Table 5 and Table 6 and Figure 6). Newly formed bone tissue was observed in all the treated cavities 60 days after the procedure. Bone tissue, which has a very good healing score (score = 6), was observed in the BGSr samples, followed by BGMgSr and BGMg (Table 4). Statistical analyses showed that there is significant difference between bone healing score of the three tested bioactive glasses (*p* = 0.025) (Table 5).

## 4. Discussion

The materials investigated in the present work, previously tested with success [11] in vitro both in terms of their bioactivity in a Simulated Body Fluid solution (SBF) and of their cytotoxicity, behave rather satisfactorily after grafting in the chosen extreme conditions.

All glasses were characterized by a pronounced ability to form hydroxycarbonate apatite (HAC) in SBF, already after one day of immersion. In fact, according to the model proposed by Hench and co-workers [21] bioactive glasses bond to bone through the formation of a HAC film on their surface, which is similar to biological apatite, i.e., the mineral phase of bone. SBF assays [22] aim to reproduce in vitro the formation of HCA, which occurs on the surface of a given bioactive material after its implantation in bone tissue. Beside their HAC-forming ability, the glasses, tested with murine long bone osteocytes (MLO-Y4) through a multi-parametric approach, were not cytotoxic. In particular, Neutral Red (NR) uptake and tetrazolium salt XTT assays were employed to evaluate cell viability, while Bromodeoxyuridine (BrdU) assay was used to investigate the possible negative effects of the samples on cell proliferation. The cytotoxicity of the glasses was studied by means of direct and indirect contact, with the aim to exclude possible cytotoxic effects of the samples’ extracts [11].

In this study, before employing these glasses as bone graft in bone regeneration of defects, we wished to investigate their capabilities in a bone defect needing a long time to repair, using critical granule-size of glasses with a high surface/volume ratio.

The rabbit was selected since it is one of the most commonly used experimental animals. It is well known that the mid-diaphyseal bone is the preferred site for transcortical bulk implants by virtue of its mechanical and biological characteristics [23], whereas metaphyseal sites are preferred for analyzing spongy bone interactions with granular materials [18]. However, there are several reasons in our study for the choice of the mid-diaphyseal femur. First of all, there were similarities of fracture toughness of this site between human and rabbits [24]. The second reason—certainly not the least important—was related to the different metabolism of long bones: the metabolic activity of the bone tissue, which is the cellular activities of bone remodeling, was greater at the metaphyseal level, intermediate at the epiphyseal level, and lower at the diaphyseal level [25]. Rabbit had faster skeletal change and bone turnover than primates [24], so an implant in mid-diaphysis could produce outcomes nearest to those achievable in man. Besides, the analysis of possible negative effects on bone processes by ions releasing from the glass dissolution could probably be better achieved in sites with a low turnover.

The routinely-used glasses are typically employed with two particle size ranges: a narrow particle size, i.e., 200–350 µm [26,27,28], and a large particle size, 500–800 µm [29,30,31], without greatly different outcomes despite the different surface/volume ratio [32]. Since in the rabbit, the amount of silicon excreted after a glass implant was particularly elevated in the first 2 months [27], we decided to decrease the size of the glass granules to speed up the ion release. Because of the small size of the granules used in this study (about 60 μm), there was a great release of ions, and this could greatly affect the bone regeneration processes.

Results not only showed that all these novel glasses did not jeopardize the bone repair, but, on the contrary and above all Sr-glasses, they induced a pronounced, greater than control, hole closure, especially for Sr-glasses. In glass-grafted defects, the new bone was formed in a statistically significant greater amount along the edge of the surgically produced hole; moreover small composites (bone/granule) were formed or new bone formations were detected inside the hole or in the medullar cavity, in close proximity to the hole, in particular when Sr-glasses were used as graft.

All the composite formations contained new granules formed by re-mineralization (precipitation) of ions coming from the glass dissolution. Such formations were particularly abundant in Sr-glasses and contained, in addition to calcium and phosphorus, also a good amount of strontium. The presence of newly formed bone surrounding the granules or in the medullar cavity, particularly in proximity to the Sr-containing granules should be pointed out; this fact seems to indicate a possible role of this element in forming material surfaces with osteoconductive effect, or to have a beneficial role on bone processes and repair.

## 5. Conclusions

In this preliminary study, for the first time, three novel bioactive glasses in powder form, each containing MgO and/or SrO, have been tested in vivo in the mid-shaft of rabbits. With respect to the powders that are typically discussed in the literature in similar works, glass granules with lower particle size (about 60 μm) were employed in order to speed up ion release.

In vivo results not only confirmed the chemical and cell culture outcomes [11], but also highlighted the absence of adverse effects on bone processes due to the glasses’ dissolution. Moreover, the novel bioactive glasses—particularly the strontium-containing compositions—had an osteoconductive effect on new bone formation, thus indicating possible beneficial effects on bone formation processes.

## Figures and Tables

**Table 1:** Compositions (in oxides mol%) of the produced glasses.

Oxides	Composition (mol%)		
	BGMg	BGSr	BGMgSr
**SiO_2_**	47.2	47.2	47.2
**P_2_O_5_**	2.6	2.6	2.6
**Na_2_O**	2.3	2.3	2.3
**K_2_O**	2.3	2.3	2.3
**CaO**	35.6	35.6	35.6
**MgO**	10	0	5
**SrO**	0	10	5

**Table 2:** Bioactive glasses implantation in rabbits’ left and right femurs.

Subject	Left Femur (Sample)	Right Femur (Sample)
**1**	BGSr	Empty hole
**2**	BGMg	Empty hole
**3**	BGMgSr	Empty hole
**4**	BGSr	BGMgSr
**5**	BGMg	BGMgSr
**6**	BGSr	BGMg

**Table 3:** Histological scoring table for bone healing used for statistical analysis.

No osteogenesis	1
Weak osteogenesis	2
Medium-low osteogenesis	3
Medium-high osteogenesis	4
Good-low osteogenesis	5
Good-high osteogenesis	6
Perfect osteogenesis	7

**Table 4:** Bone healing scores for bioactive glasses implanted in rabbits’ left and right femurs. (see Table 2 for the list of materials).

Days 60		
Subject	Left Femur (Bone Healing Score)	Right Femur (Bone Healing Score)
**1**	6	2
**2**	5	2
**3**	4	2
**4**	6	3
**5**	3	4
**6**	5	3

**Table 5:** Kruskal–Wallis results in terms of bone healing scores (BHS) for BGMg, BGSr, BGMgSr bioactive glasses, and sham hole (empty).

Material		N	Mean Rank	Test Statistics a,b		
				Chi Square	df	Asymp.Sig.
BHS	1 BGMg	3	6.50	9.373	3	0.025
	2 BgMgSr	3	6.67			
	3 BgSr	3	10.83			
	4 Empty hole	3	2.00			
	Total	12				

^a^ Kruskal–Wallis Test; ^b^ Grouping Variable Material.

**Table 6:** Amount of newly formed bone present inside the osteotomic gap expressed as BV/TV% (histomorphometric parameter).

Sample	TV (mm^2^)	BV/TV %
BGMg	4502	49,797
BGSr	5003	54,638
BGMgSr	3128	50,596
